# Association of HIV, cardiovascular risk factors, and carotid intimal media thickness: A cross-sectional study in Western Kenya

**DOI:** 10.1097/MD.0000000000031366

**Published:** 2022-11-25

**Authors:** Maritza T. Farrant, Sarah J. Masyuko, John Kinuthia, Alfred O. Osoti, Jerusha N. Mogaka, Tecla M. Temu, Jerry S. Zifodya, Damalie Nakanjako, Faith Ameda, Carey Farquhar, Stephanie T. Page

**Affiliations:** a Department of Global Health, University of Washington, Seattle, WA, USA; b Ministry of Health, Nairobi, Kenya; c Department of Research and Programs, Kenyatta National Hospital, Nairobi, Kenya; d Department of Obstetrics and Gynaecology, University of Nairobi, Nairobi, Kenya, Tulane University, New Orleans, LA, USA; e Department of Medicine, Section of Pulmonary, Critical Care & Environmental Medicine, Tulane University, New Orleans, LA, USA; f Department of Medicine, School of Medicine, Makerere University College of Health Sciences, Kampala, Uganda; g Department of Radiology, College of Health Sciences, Makerere University, Kampala, Uganda; h Department of Medicine, University of Washington, Seattle, WA, USA; i Department of Epidemiology, University of Washington, Seattle, WA, USA.

**Keywords:** Cardiovascular risk factors, carotid intimal media thickness, disease, human immunodeficiency virus, Western Kenya

## Abstract

The carotid intimal media thickness (CIMT) is a validated measure of subclinical atherosclerosis. Human immunodeficiency virus (HIV) is a risk factor for cardiovascular disease (CVD) and has been associated with CIMT in North America and Europe; however, there are limited data from Sub-Saharan Africa (SSA). In this cross-sectional study, we measured CIMT in a cohort of 262 people living with HIV (PLHIV) on antiretroviral therapy (ART) for ≥6 months and HIV-negative adults in western Kenya. Using linear regression, we examined the associations between CVD risk factors and CIMT, both overall and stratified according to the HIV status. Among the PLHIV, we examined the association between CIMT and HIV-related factors. Of 262 participants, approximately half were women. The HIV-negative group had a higher prevalence of age ≥55 years (*P* = .002), previously diagnosed hypertension (*P* = .02), treatment for hypertension (*P* = .03), and elevated blood pressure (BP) (*P* = .01). Overall prevalence of carotid plaques was low (15/262 [6.0%]). HIV-positive status was not significantly associated with a greater mean CIMT (*P* = .19). In multivariable regression models, PLHIV with elevated blood pressure or treatment for hypertension had a greater mean CIMT (*P* = .002). However, the CD4 count, viral load, and ART regimen were not associated with differences in CIMT. In the HIV-negative group, older age (*P* = .006), high total cholesterol levels (*P* = .01), and diabetes (*P* = .02) were associated with a greater mean CIMT. In this cross-sectional study of Kenyan adults, traditional CVD risk factors were found to be more prevalent among HIV-negative participants. After multivariable regression analysis, we found no association between HIV status and CIMT, and PLHIV had fewer CVD risk factors associated with CIMT than HIV-negative participants did. HIV-specific factors were not associated with the CIMT.

## 1. Introduction

Human immunodeficiency virus (HIV) infection is associated with a nearly two-fold increased risk of cardiovascular disease (CVD).^[1,2]^ Contributing factors include increased chronic inflammation and immune activation associated with HIV infection,^[3,4]^ traditional CVD risk factors, and antiretroviral therapy (ART), particularly regimens including protease inhibitors (PIs).^[1,5]^ Worldwide, there are now a greater number of people living with HIV (PLHIV), with the majority living in Sub-Saharan Africa (SSA).^[6,7]^ Given the large and increasing global burden of CVD,^[7]^ clinical tools are needed to improve risk stratification and management of CVD for PLHIV in SSA, which is reliable, feasible, and cost-effective.

In longitudinal studies in North America and Europe, carotid intimal media thickness (CIMT) has been validated as an indicator of subclinical atherosclerosis and a predictor of future CVD events, such as stroke and myocardial infarction (MI).^[8,9]^ Carotid plaque, a focal lesion in the arterial wall that can impede blood flow, is also recognized as a marker of subclinical atherosclerosis and a predictor of CVD independent of CIMT.^[10]^ CIMT varies by age, race, and sex; however, carotid plaques may be present even when CIMT does not increase.^[11–13]^ Both CIMT and the presence of carotid artery plaque can be assessed noninvasively using ultrasound. This provides an advantage for screening for the presence of subclinical CVD over more invasive and costly procedures, such as coronary angiography.

In a large meta-analysis and systematic review of observational studies that included PLHIV, a higher CIMT was associated with HIV-positive status.^[2,14,15]^ However, most of these studies were from regions with low HIV prevalence, such as Europe or North America, and greater use of protease inhibitors (PIs) than other antiretroviral drugs.^[4,14]^ In a small number of cross-sectional studies from SSA, an association between PLHIV and greater CIMT has not been uniformly observed. Thus, given the higher prevalence of HIV in SSA as well as the different environmental and genetic factors that confer risk for CVD, more work is needed to determine the relationships between abnormal CIMT, HIV, and CVD risk factors in SSA to efficiently target interventions for individuals at the highest risk.

In this study, we evaluated the association between HIV status and CIMT in a rural setting in western Kenya with a high HIV prevalence. We also examined the association between CVD risk factors and CIMT when stratified by HIV status. Finally, we examined the association between CIMT and HIV-specific factors such as HIV viral load and CD4 count.

## 2. Methods

### 2.1. Study design and setting

The parent study enrolled PLHIV-and HIV-negative men and women living in western Kenya and was conducted in collaboration with the University of Washington, University of Nairobi, Kenyatta National Hospital, and Kenya Ministry of Health. Participants were enrolled at the Kisumu Sub-County Hospital in Kisumu County, which serves the surrounding communities in Western Kenya. The main study matched participants according to HIV status and sex, enrolling 150 PLHIV men, 150 PLHIV women, 149 HIV-negative men, and 149 HIV-negative women. The eligibility criteria were being at least 30 years old and living within a 50 km radius of the hospital. PLHIV had to be enrolled and regularly attended the HIV comprehensive care clinic and used ART for a minimum of six months. PLHIV who met the inclusion criteria and consented to participate in the study were enrolled by a comprehensive care clinic study nurse. HIV-negative participants were recruited from hospital HIV testing sites until the required sample size was achieved.

Human subject approval was obtained from the University of Washington Institutional Review Board and the Kenyatta National Hospital/University of Nairobi Ethical and Scientific Review Committee. All the participants provided written informed consent.

### 2.2. Study procedures

The detailed procedures for this cross-sectional study have been published.^[16]^ We collected subject health and demographic data using the World Health Organization STEP wise approach to Surveillance (STEPS) questionnaire^[17]^ modified for the Kenyan context. This included a self-reported history of cardiovascular risk factors and physical examination. Blood pressure (BP) was recorded twice in each arm, five minutes apart, using a digital sphygmomanometer (CH 453, Omron Health). Anthropometric measurements included height, weight, and waist circumference. Participants returned after an initial visit for carotid ultrasound and fasting blood tests for lipid, glucose, and HIV-specific factors such as viral load and CD4 count.

### 2.3. CIMT ultrasound examination

Radiographers at Kisumu County Referral Hospital received training in scanning techniques, acquisition, and interpretation of images using a Sonosite M Turbo ultrasound machine (FUJIFILM, Sonosite Inc., Bothell) with a HFL38X/13-6 MHZ transducer. Training and quality assurance for radiographers was provided by FM, a radiologist experienced in performing carotid ultrasound and measurement of CIMT for research purposes.^[18]^ The standard operating procedures for this study were based on protocols for the measurement of CIMT from the American Society of Echocardiography.^[19]^ The Sonosite software program (SonoCalc V5.0.0.12) was used for image acquisition and reporting. Intrareader reliability was assessed during training, but not made available.

Examination of the extracranial carotid arteries included identification of carotid plaques and measurement of CIMT in both the right and left common carotid arteries. First, the study participant was positioned in a semi-upright position with the head slightly tilted to the left or right depending on the side being examined. The common carotid artery (CCA) was identified with the ultrasound probe in the longitudinal position using a proximal point 10 mm from the carotid bulb. Measurements of intima-medial thickness were taken with the ultrasound probe in the anterior, posterior, and lateral positions, and three measurements of the mean IMT segment, calculated automatically by the software, were made at the near and far vessel walls (6 in total). The combined average of all measurements was reported as the overall CIMT for the common carotid artery in a similar study.^[18]^ A mean CCA IMT >1.5 mm at any of the measured points was considered a plaque.

Ultrasound images of each participant were recorded, stored, and transferred after each examination. The research radiologist assigned to training (FM) performed the subsequent reading and reporting of the studies, and a formal report was generated using SonoCalc V5.0.0.12. In total, only 262 participants had a formal report verified for inclusion in the final analysis due to funding limitations. These participants were selected via consecutive sampling and matched by age, sex, and HIV status. The smaller sample size was calculated to be adequately powered to detect significant differences in the CIMT.

### 2.4. Definition of dependent and independent variables

Independent variables included the following demographic and lifestyle factors: age, sex (male or female), smoking status (current = smoking within 12 months and ex-smoker ≥12 months without use), and educational level (no formal schooling, primary schooling, secondary school completed, or more than secondary school). CVD history included a self-reported prior diagnosis of hypertension (HTN), HTN treatment, diabetes treatment, and prior MI or stroke. We classified hypertension according to the Kenyan hypertension guidelines adopted from the Eighth Joint National Committee on Prevention, Detection, Evaluation, and Treatment of High Blood Pressure (JNC VIII).^[20]^ We used the Consensus Criteria 2009 for metabolic syndrome (MetS)^[21]^ to define the following: elevated total cholesterol (TC) > 200 mg/dL, elevated low-density lipoprotein (LDL-C) > 130 mg/dL, reduced high-density lipoprotein (HDL-C) (for women < 50 mg/dL and for men < 40 mg/dL), elevated triglycerides (TG) > 150 mg/dL, and abdominal obesity (waist circumference > 88 cm for women and > 94 cm for men). We defined diabetes as a fasting blood glucose level ≥ 126 mg/dL or use of oral hypoglycemic agents, as per the World Health Organization recommendations.^[22]^ HIV status as an independent variable, as well as duration of ART (years), current CD4 count (cells/mm^3^), nadir CD4 count (cells/mm^3^), suppressed viral load (<1000 copies/mL), undetectable viral load (<50 copies/mL), and ART regimen (either protease/first-line vs non-protease inhibitor/second-line regimen). CIMT was analyzed as a continuous dependent variable.

### 2.5. Sample size and statistical analysis

This study was powered to detect a minimum mean CIMT difference of.04 mm with 80% confidence enrolling a minimum of 110 PLHIV and 110 HIV-negative participants. This reference mean difference in CIMT was also used in another SSA study^[23]^ and reported in a large systematic review of observational studies^[14]^ and in a later study that compared PLHIV in the fat redistribution and metabolic changes in HIV infection (FRAM) study with HIV-negative patients in the Multi-Ethnic Study of Atherosclerosis (MESA).^[15,24]^

Categorical baseline characteristics were compared between PLHIV and HIV-negative individuals using the chi-squared or Fisher’s exact tests. Given the small sample size in each subgroup, we classified age, education, and body mass index (BMI) as binary variables: age (<55 vs ≥55 years), education level (below or above the secondary level), and BMI (<25 or ≥25 kg/m^2^). The blood pressure was calculated as the average of all readings. CIMT was analyzed as a continuous variable and was normally distributed. Linear regression was used to assess the association between independent variables and CIMT for the entire cohort, which was then stratified by HIV status. Residual plots were used to assess the validity of these four assumptions. There were 21 outliers in the CIMT dataset. These outliers were validated with the corresponding CIMT reports and were not excluded as they were not influential. Missing data were excluded from analysis.

Initially, simple linear regression models were used to assess the association between an independent variable and CIMT, first with the entire cohort, and then stratified by HIV status. In the multivariable regression model, the independent variables (*P* ≤ .15) in the simple linear regression models were included, as well as variables of clinical significance that a priori included age, sex, and education status. The multivariable model was conducted with backward regression and stepwise removal of variables, until the best model fit was obtained. Statistical significance was set at *P* < .05. All statistical analyses were conducted using the R software (version 3.6.1 [RStudio Team (2020]. RStudio: Integrated Development for R. RStudio, PBC, Boston, MA).

## 3. Results

### 3.1. Demographic and clinical characteristics

Of the 262 participants, 145 were PLHIV negative and 117 were HIV negative (Table 1). The proportion of those ≥55 years was lower in the PLHIV than in the negative group, 40% versus 59%, respectively (*P* = .02). Women comprised over half of the PLHIV and less than half of the HIV-negative group; 56% versus 46% (*P* = .12). The proportion reporting secondary education or higher in both groups was similar, 40% versus 42% (*P* = .76).

**Table 1:** Baseline characteristics of participants by HIV status.

Variables	Total N = 262	PLHIV, N = 145	HIV neg, N = 117	*P* value
	n (%)	n (%)	n (%)	
**Demographic variables**				
Age ≥55 yr	**126 (48.1**)	**57 (39.3**)	**69 (59.0**)	**.002**
Women	135 (51.5)	81 (55.9)	54 (46.2)	.12
Secondary education and above	107 (40.8)	58 (40.0)	49 (41.9)	.76
**CVD history**				
Prior stroke or MI	8 (3.1)	6 (4.1)	2 (1.8)	.30
Hypertension	**39 (14.9**)	**15 (10.3**)	**24 (20.5**)	**.02**
Hypertension treatment	**14 (5.3**)	**5 (3.4**)	**9 (7.7**)	**.03**
Diabetes treatment	2 (.01)	2 (.03)	0	
**Lifestyle factors**				
Current smoker (<12 mo)	13 (5)	5 (3.4)	8 (6.8)	.26
Ex-smoker	31 (11.8)	16 (11.0)	15 (12.8)	.65
**Physical exam**				
Elevated BP	**64 (24.4**)	**27 (18.6**)	**37 (33.3**)	**.01**
Anthropometric measures				
Elevated BMI	88 (33.6)	47 (32.4)	41 (35.0)	.65
Abdominal obesity	65 (24.8)	33 (22.8)	32 (27.3)	.39
**Laboratory measurements** *				
Elevated TC	56 (22.8)	31 (22.3)	25 (23.4)	.84
Elevated LDL-C	45 (18.3)	22 (15.9)	23 (21.5)	.22
Reduced HDL-C	70 (28.6)	33 (23.9)	37 (34.5)	.06
Elevated TG	**23 (9.4**)	**18 (12.9**)	**5 (4.7**)	**.03**
Diabetes	6 (2.4)	2 (1.4)	4 (3.7)	.41
**HIV-specific factors**				
Duration on ART (yr)		9 (4.0–11.0)		
Current CD4 count (cells/mm^3^)		489.0 (343.0–654.0)		
Nadir CD4 count (cells/mm^3^)†		372 (215–546)		
VL suppressed‡		139 (100)		
Undetectable VL		112 (8.2)		
**On ART**				
1st line regimen (NNRTI-PI)		121 (83.4)		
2nd line regimen (PI)		24 (16.6)		

Definitions: BP ≥ 140 mm Hg or ≥ 90 mm Hg. Elevated BMI (≥ 25 kg/m2). Abdominal obesity = waist circumference > 88 cm for women and > 94 cm for men. Elevated TC > 200mg/dL. Elevated LDL-C > 130 mg/dL. Reduced HDL-C < 50 mg/dL in women and < 40 mg/dL in men. Elevated TG level ≥ 150 mg/dL. Diabetes = fasting glucose ≥ 126 mg/dL. HIV related factors; Suppressed VL < 1000copies/mL; Undetectable VL < 50 copies/mL.

ART = antiretroviral therapy, BMI = body mass index, BP = blood pressure, CVD = cardiovascular disease, DBP = diastolic blood pressure, HDL = high-density lipoprotein, HIV = human immunodeficiency virus, HTN = hypertension, IQR = interquartile range, LDL = low-density lipoprotein, MI = myocardial infarction, NNRTI = non-nucleoside reverse transcriptase inhibitor, PI = protease inhibitor, PLHIV = people living with HIV, SBP = systolic blood pressure, TGs = triglyceride, VL = viral load.

* 16 missing values for laboratory measurements: 6 in the PLHIV group and 10 in the HIV-negative group.

† 8 missing observations for nadir CD4 count.

‡ 6 missing observations for viral load measurements.

Few participants had established CVD; overall, only 3% of participants reported prior stroke or MI. Approximately 10% of the PLHIV reported a prior diagnosis of HTN compared to 21% in the HIV-negative group (*P* = .02); however, few in either group reported taking an antihypertensive medication (3% vs 8%; *P* = .09). Only one participant reported taking aspirin and no participant was taking statins. The use of tobacco products (cigarettes, manufactured or handrolled cigarettes, cigars, waterpipes [shisha], or pipes [kiko]) in the last 12 months was low, with only 3% among PLHIV and 7% in the HIV-negative group reporting use (*P* = .24). The proportion of those reporting past use of tobacco products in each group was also low: 11% in PLHIV and 13% in the HIV-negative group (*P* = .33).

The proportion of participants with elevated blood pressure was higher in the HIV-negative group than that in the PLHIV group (*P* = .02). Approximately one-third of the patients in each group had elevated BMI (*P* = .65). The proportion of participants with abdominal obesity did not differ significantly between groups (*P* = .39).

With respect to laboratory measurements, less than one-third of participants had dyslipidemia. There was no significant difference in the proportion of participants with elevated TC levels between PLHIV-and negative and HIV negative participants (*P* = .22). However, the proportion of patients with elevated TG levels was higher in the PLHIV group (*P* = .03). The proportion of patients with lower HDL-C levels in the PLHIV group was significantly lower than that in the HIV-negative group (*P* = .06). There was no difference between PLHIV and HIV-negative in those diagnosed with diabetes, and the percentages were very low (1.4% vs 3.7%, *P* = .41). Only two participants reported receiving diabetes treatment among the PLHIV, including insulin (1) and an oral agent (1).

Among PLWH, the median duration of ART was 9.0 years (interquartile range [IQR] 4.0–11.0). The median CD4 count was 489 cells/mm^3^ (IQR 343–654) and the median nadir CD4 count was 372 cells/mm^3^ (IQR 215–546). All 139 participants with available samples were virally suppressed, with a viral load of <1000 copies/mL. Approximately 80% (112/145) had undetectable viral loads, and 121/145 (84%) were on a first line or non-nucleoside reverse transcriptase inhibitor (NNRTI) regimen, with 24 (16%) on a second line or protease inhibitor (PI) regimen.

### 3.2. CIMT measurements

The overall mean CIMT in the cohort was 0.42 mm (standard deviation [SD] 0.12). The mean CIMT was lower in the PLHIV group than in the HIV-negative group (0.41 mm [SD.12] vs 0.44 mm [SD 0.14], *P* = .02) (Table 2). Only a few participants had CIMT thickness of ≥0.78 mm (4/262, 2.0%), the upper limit of what has been defined as normal in a longitudinal non-African cohort,^[25]^ and used in other SSA studies as a marker of subclinical atherosclerosis.^[18]^ We also noted the presence of plaque, a measure of subclinical atherosclerosis, in 6% (15/262) of participants, nine in PLHIV, and six in the HIV-negative participants (*P* = .71). The Plaques were mostly located in the carotid bulb (13/15, 87%).

**Table 2:** Factors associated with differences in carotid intimal media thickness (CIMT) among all study participants who underwent carotid ultrasound (n = 262).*,†

	Simple linear regression*		Multivariable linear regression†	
Variables	β coefficient (95% CI)	*P* value	β coefficient (95% CI)	*P* value
**HIV positive status**	−0.03 (−0.07,−0.01)	.02	−0.02 (−0.05,0.01)	.19
**Demographic variables**				
≥55 yr	0.05 (0.01,0.07)	.004	0.03 (0.001,0.07)	.04
Female sex	0.02 (−0.01,0.05)	.24		
**Lifestyle factors**				
Current smoker	−0.01 (−0.08,0.06)	.76		
Ex-smoker	0.04 (−0.01,0.08)	.12	0.04 (−0.01,0.08)	.15
Secondary education or above	−0.01 (−0.05,0.01)	.26		
**CVD history**				
Hypertension	0.03 (−0.01,0.07)	.15	−0.01 (−0.05,0.04)	.83
**Physical exam**				
Elevated BP or on treatment	0.05 (0.01,0.08)	.01	0.04 (−0.005,0.07)	.09
**Anthropometric factors**				
Elevated BMI	−0.01 (−0.04,0.02)	.40		
Abdominal obesity (females)	0.01 (−0.03,0.05)	.63		
Abdominal obesity (males)	−0.03 (−0.10,0.04)	.34		
**Laboratory measurement**				
Elevated TC	0.03 (−0.01,0.07)	.10	−0.03 (−0.06,0.06)	.99
Elevated LDL-C	0.04 (−0.001,0.08)	.06	0.03 (−0.02,0.07)	.22
Reduced HDL- C (females)	−0.004 (−0.05,0.04)	.87		
Reduced HDL- C (males)	−0.03 (−0.08,0.03)	.38		
Elevated TG	−0.04 (−0.06,0.05)	.89		
Diabetes	0.12 (0.02,0.22)	.02	0.11 (0.01,0.21)	.02

Definitions: Elevated BP ≥140 mm Hg or ≥90 mm Hg. Elevated BMI (≥25 kg/m^2^), abdominal obesity = waist circumference >88 cm for women and >94 cm for men. Elevated TC > 200 mg/dL. Elevated LDL-C >130 mg/dL. Reduced HDL-C <50 mg/dL in women and <40 mg/dL in men. Elevated TG ≥ 150 mg//dL, diabetes (WHO) = fasting glucose ≥126 mg/dL, or hypoglycemic agents.

BMI = body mass index, BP = blood pressure, CVD = cardiovascular disease, DBP = diastolic blood pressure, HDL = high-density lipoprotein, HIV = human immunodeficiency virus, HTN = hypertension, LDL = low-density lipoprotein, MI = myocardial infarction, SBP = systolic blood pressure, TGs = triglyceride.

* Independent variables included in the multivariable regression model with *P* ≤ .15.

† The final multivariable linear regression model after backward stepwise removal of independent variables.

## 4. Traditional CVD risk factors and CIMT in the cohort

### 4.1. Simple linear regression analyses

Overall, several factors were significantly associated with CIMT in simple linear regression models using data from the entire cohort, including HIV status, older age, elevated blood pressure and/or use of antihypertensive agents, and diabetes (Table 2).

HIV positive status was associated with a 0.03 mm lower CIMT (95% confidence interval [CI]: −0.01, 0.07) compared to those who were HIV-negative (*P* = .02). Older age, ≥55 years) was associated with a of 0.05 mm higher CIMT (95% CI: 0.01, 0.07), compared to those <55 years of age (*P* = .004). Those with elevated LDL-C had a 0.04 mm higher mean CIMT, compared to those with a normal range LDL-C (95%CI: −0.001, 0.08, *P* = .06), although this was borderline in terms of statistical significance. Finally, a diagnosis of diabetes was associated with a 0.12 mm higher mean CIMT in comparison to those without a diagnosis of diabetes (95%CI: 0.02, 0.22, *P* = .02).

### 4.2. Multivariable regression analysis

The independent variables in the model^*^ included older age, female sex, secondary education, ex-smoking status, high blood pressure or hypertension treatment, elevated TC and LDL-C levels, and diabetes (Table 2). After adjustment, HIV-positive status was not significantly associated with greater CIMT (*P* = .19). Significant associations^†^ were seen in those who were older with a 0.03 mm higher CIMT (95%CI: 0.001, 0.07, *P* = .04) and in those with a diagnosis of diabetes with a 0.11 mm higher CIMT, compared to those without diabetes (95%CI: 0.008, 0.21, *P* = .02).

## 5. Traditional CVD risk factors and CIMT stratified by HIV status

### 5.1. Simple linear regression analysis

Among PLHIV (Table 3), those with elevated blood pressure or on HTN treatment had a 0.06 mm higher CIMT compared to those with a normal range blood pressure or not on treatment (95%CI: 0.02, 0.11, *P* = .003). None of the HIV-related factors were significantly associated with CIMT in the simple linear regression analysis.

**Table 3:** Factors associated with differences in CMIT stratified by HIV status with simple and multivariable regression.*^,^†

	PLHIV (n = 145)*		HIV-neg (n = 117)†	
	β coefficient (95%CI)	*P* value	β coefficient (95%CI)	*P* value
**Demographic variables**				
Age ≥55 yr	0.01 (−0.02,0.05)	.50	0.07 (0.02,0.12)	.006**
Female sex	0.03 (−0.006,0.07)	.10	0.01 (−0.04,0.06)	.66
**Lifestyle factors**				
Current smoker	−0.02 (−0.12,0.08)	.65	−0.01 (−0.11,0.09)	.78
Ex-smoker	0.04 (−0.02,0.09)	.23	0.03 (−0.04,0.11)	.36
Secondary education	−0.02 (−0.06,0.02)	.26	−0.01 (−0.06,0.04)	.57
**CVD history**				
Hypertension	0.03 (−0.03,0.09)	.36	0.02 (−0.04,0.09)	.49
**Physical exam**				
Elevated BP or on treatment	0.06 (0.02,0.11)	.003*	0.01 (−0.04,0.07)	.59
**Anthropometric factors**				
Elevated BMI	0.004 (−0.04,0.04)	.84	−0.03 (−0.09,0.02)	.16
Abdominal obesity (females)	0.01 (−0.04,0.06)	.81	0.01 (−0.07,0.08)	.87
Abdominal obesity (males)	−0.002 (−0.09,0.09)	.96	−0.06 (−0.17,0.04)	.23
**Laboratory measurement**				
Elevated TC	−0.01 (−0.05,0.04)	.72	0.08 (0.02,0.14)	.01**
Elevated LDL-C	0.01 (−0.05,0.06)	.81	0.07 (0.005,0.13)	.04
Reduced HDL-C(females)	−0.004 (−0.05,0.04)	.87	−0.04 (−0.12,0.04)	.28
Reduced HDL-C (males)	0.002 (−0.08,0.08)	.95	−0.06 (−0.14,0.02)	.16
Elevated TG	−0.02 (−0.08,0.04)	.52	0.08 (−0.05,0.20)	.21
Diabetes	0.01 (−0.15,0.17)	.90	0.17 (0.03,0.31)	.02**
**HIV related factors**				
Duration on ART (yr)	0.0004 (−0.004,0.004)	.86		
CD4 count (current)	0.005 (−0.04,0.05)	.79		
CD4 count (nadir)	−0.003 (−0.04,0.03)	.86		
Undetectable VL	−0.02 (−0.06,0.02)	.38		
ART 2nd line regimen (PI)	0.003 (−0.05,0.05)	.88		

Definitions: elevated BP ≥ 140 mm Hg or ≥ 90 mm Hg. Elevated BMI (≥25 kg/m^2^), abdominal obesity = waist circumference >88 cm for women and >94 cm for men. Elevated TC >200 mg/dL. Elevated LDL-C >130 mg/dL and elevated TG ≥150 mg/dL. Reduced HDL-C <50 mg/dL in women and <40 mg/dL in men. Diabetes (WHO) was defined as fasting glucose ≥126 mg/dL or hypoglycemic agents. HIV related factors; Suppressed VL < 1000 copies/mL; Undetectable VL <50 copies/mL.

95% CI = 95% confidence interval, ART = antiretroviral therapy, BMI = body mass index, BP = blood pressure, CD4 = cluster differentiation 4, CVD = cardiovascular disease, DBP = diastolic blood pressure, HDL = high-density lipoprotein, HIV = human immunodeficiency virus, HTN = hypertension, LDL = low-density lipoprotein, MI = myocardial infarction, PI = protease inhibitor, SBP = systolic blood pressure, TGs = triglyceride.

* Final regression model for PLHIV: female sex; β coefficient =0.03 ([95%CI: −0.004,0.07], *P* = .08); elevated BP or on HTN treatment β coefficient =0.07 ([95%CI:0.02,0.11], *P* = .002*). *Indicates significance in the final model and not shown in table.

† Final regression model for HIV-negative: older age; β coefficient =0.07 ([95% CI:0.01,0.12], *P* = .01**); elevated BP or on HTN treatment;β coefficient = −0.04 ([95%CI: −0.09,0.15], *P* = .14); high TC; β coefficient =0.09 ([95%CI:0.03,0.14], *P* = .01**); diabetes; β coefficient =0.21 ([95% CI: 0.08,0.34], *P* = .002**). **Indicates those that were significant in the final model and not shown in table.

Several CVD risk factors were associated with a higher CIMT in the HIV-negative group (Table 3). Older age was associated with a 0.07 mm higher mean CIMT (95%CI: 0.02, 0.12, *P* = .006), those with elevated TC had a 0.08 mm higher mean CIMT (95%CI: 0.02, 0.14, *P* = .01), and those with an elevated LDL-C had a 0.07 mm higher mean CIMT (95%CI: 0.005, 0.13, *P* = .04). Finally, those with diabetes had a 0.17 mm higher mean CIMT (95%CI: 0.03, 0.31, *P* = .02).

### 5.2. Multivariable linear regression analysis

Among PLHIV (Table 3), the model included older age, female sex, secondary school education, high BP, and use of hypertension treatment. After adjustment, the final model^*^ only included those with elevated blood pressure or hypertension treatment that was associated with a 0.07 mm higher mean CIMT (95%CI: 0.02, 0.11, *P* = .002). Female sex was not significantly associated with CIMT in the final model (*P* = .08).

In the HIV-negative group (Table 3), the model included older age, female sex, secondary school education, elevated TC and LDL-C levels, and diabetes mellitus. After adjustment, the final model^†^ included only older age, elevated TC level, and diabetes. Those Participants who were older had a 0.07 mm higher mean CIMT (95%CI: 0.02, 0.12, *P* = .006), those with elevated TC had a 0.09 mm higher CIMT (95%CI: 0.03, 0.14, *P* = .01), and those with diabetes had a 0.17 mm higher mean CIMT (95%CI: 0.03, 0.31, *P* = .02).

## 6. Discussion

In this cross-sectional study of Kenyan adults, with a virally suppressed group of PLHIV and a community-matched group of HIV-negative individuals, HIV-positive status was not associated with a higher CIMT after adjusting for other significant CVD risk factors. This lack of association has also been observed in several other studies conducted in SSA where CIMT was measured^[23,26–28]^ and is contrary to findings from studies in high-and middle-income countries that found an association between HIV infection and greater CIMT.^[29–31]^ Overall, our CIMT values were lower in this cohort than in similar SSA studies.^[23,26,27,32]^ Importantly, we found that some CVD risk factors were associated with higher CIMT among HIV-negative participants, and our results are consistent with other SSA studies that have shown that PLHIV with sustained viral suppression do not show a difference in CIMT, as well as from other low-middle-income countries (LMICs)^[32,33]^ suggesting that well-controlled HIV infection may not be a significant risk factor for early CVD in SSA. This also supports the need for longitudinal studies to better understand the relationships among HIV, CVD risk factors, and CVD morbidity, specifically in SSA, to support targeted interventions.

Our study findings in PLHIV compare to a study by Muiru et al (2018) that reported a significantly lower mean CIMT in PLHIV than in HIV-negative patients (0.62 mm vs 68 mm).^[34]^ PLHIV also had lower CVD risk scores, smoking prevalence, and median systolic blood pressure in this cohort. The four center H3 Africa-HW1 Gen Study^[26]^ reported an overall age and sex mean adjusted CIMT of.64 mm with a statistically different mean CIMT between centers: the highest CIMT at the Ghana site and lowest in Agincourt, one of the South African (SA) sites (0.69 mm vs 60 mm). The prevalence of HIV in the H3 study varied from <1% to 35%. HIV status was self-reported in some centers but was associated with lower CIMT along with high HDL-C and current alcohol consumption. After adjustment for age, education status, education status, and traditional CVD risk factors in the pooled analysis.^[26]^ We observed a lower overall mean CIMT than these studies and low overall plaque prevalence that did not differ by HIV status. Our results are similar to the Ndlovu cohort, which reported a carotid plaque prevalence of 4.5% that did not differ by HIV status,^[27]^ but different from a Botswana study, which reported a higher plaque prevalence in PLHIV (15% in PLHIV vs 7% in HIV-negative persons).^[23]^ This may reflect better management of cardiovascular risk factors in SSA countries, or other lifestyle or environmental factors that were not measured in this study.

In addition, the measurement of CIMT and definition of subclinical atherosclerosis vary across studies. Some have defined subclinical atherosclerosis as a CIMT ≥0.78 mm,^[18,32,35,36]^ a criterion derived from a European longitudinal study tracking intimal media thickness of children with familial hypercholesterolemia and healthy comparators.^[25]^ We did not use this definition because it has not been validated in SSA and very few participants had a CIMT above this threshold. Additional factors that may make study comparisons difficult include differing ultrasound scan techniques, sex distribution, study size, and the use of different measures, such as maximum CIMT rather than the overall mean in some cohorts.^[31,37]^

In our study, we observed a greater prevalence of CVD risk factors in the HIV-negative group, notably older age, a history of hypertension, hypertension treatment, and elevated blood pressure. This was also true in the main study^[16]^ which included younger individuals, where the prevalence of MetS was found to be higher overall and greater in HIV-negative adults, suggesting an increased risk of future CVD events in this group. A higher prevalence of CVD risk factors in HIV-negative study populations has been observed in other studies conducted in SSA, including recent cross-sectional studies in South Africa.^[27,28]^ We also observed that elevated triglyceride levels were more prevalent in PLHIV. Hypertriglyceridemia in PLHIV has also been observed in other studies^[38]^ including a large pooled analysis of 21,000 participants from SSA that observed a two-fold higher probability of high TGs levels in those on ART.^[39]^

We also observed several cardiovascular risk factors in the cohort that were positively associated with CIMT in the multivariable linear regression models that differed when stratified by HIV status. In PLHIV, elevated blood pressure and use of hypertensive treatment were significantly associated with higher CIMT in the multivariable regression model. In the HIV-negative group, older age, high TC levels, and diabetes were associated with a higher CIMT. These findings have also been observed in other SSA studies and in studies of low-to middle-income countries.^[23,34,35]^ Interestingly, elevated blood pressure was not positively associated with CIMT in this group despite the significantly higher prevalence of reported HTN (*P* = .02). However, self-reported use of hypertension treatment was also higher (*P* = .03) than in PLHIV.

Of note, our finding of a positive association between elevated blood pressure and hypertension treatment with CIMT in the PLHIV group in the multivariable regression model was interesting given the younger median age, lower prevalence of self-reported hypertension, hypertension treatment, and lower prevalence of elevated blood pressure compared to the HIV-negative group. Smoking prevalence was low overall, and we did not find a significant difference in the prevalence of higher BMI, central obesity, or dyslipidemia between the groups, except for the higher prevalence of hypertriglyceridemia in PLHIV. A possible explanation may be that PLHIV may have increased markers of endothelial dysfunction despite longstanding viral suppression.^[28,40–43]^ This may contribute to a higher CIMT in the context of under-reporting of hypertension treatment and BP status. Measurement of inflammatory markers such as IL-6 and C-reactive protein might further support this hypothesis.

We did not observe an association between ART and CIMT. The lack of association has also been observed in some other SSA and LMIC studies,^[23,35]^ but a more recent SSA study found an association when stratified by age between the time spent on PLHIV on ART^[27]^ and the HIV-negative study group (in those >30 years of age).

Finally, the findings of the association of fewer CVD risk factors with higher CIMT in PLHIV in this study suggest that PLHIV may have better access to and engagement in medical care and education on lifestyle modification within the HIV comprehensive care system (), highlighting the importance of primary prevention of CVD risk factors in the general population. It should be noted that the use of statins and aspirin, the mainstays of primary prevention of CVD in high-income countries, was almost nonexistent across the cohort.

The strengths of this study include the use of a HIV-negative comparator group as a baseline to assess the relationship between CVD risk factors and CIMT in a community-based cohort. We also used validated measures of the carotid artery to obtain CIMT measurements.

Limitations include the cross-sectional study design, which does not permit analysis of the progression of subclinical atherosclerosis and the effect of treatment of CVD risk factors and HIV disease management. Following the study, we were unable to review the scans independently to ascertain intra and inter-reader reliability due to technical issues which was also a limitation. Self-reporting of cardiovascular risk factors, such as a history of hypertension and diabetes, as well as the use of medications to treat these conditions, was a further limitation of this analysis. Although this study was appropriately powered to detect significant differences in CIMT, it was smaller than the original cohort, and despite matching by age, sex and HIV status, the HIV-negative group is older. However, we adjusted for this in the analysis and did not see a significant difference in CIMT.

In our study, we did not observe a significant association between HIV status and CIMT, after adjusting for other known CVD risk factors. We observed a higher prevalence of some CVD risk factors in the HIV-negative group, and associations between some of these CVD risk factors and CIMT. These findings are supported by other SSA studies that observed more significant associations between CVD risk factors and higher CIMT in HIV-negative groups and may reflect better access to medical care and support for PLHIV in SSA. Our CIMT measurements and plaque prevalence were lower than those in other studies and may be due to differing study population factors, sampling techniques, study design and/or CIMT quantification techniques. However, inclusion of more participants in our cohort with a longer follow up period might demonstrate results that are similar to other SSA studies with respect to CIMT.

Although there are some limitations to the study design, these results add to the body of literature, highlighting the importance of the primary prevention of CVD in the general population. Identifying the most consistent and powerful tool to quantify the CVD risk for PLHIV in SSA will require larger studies with longitudinal follow-up and documentation of cardiovascular events. CIMT, when used in conjunction with traditional markers for CVD, may be a useful tool in an LMIC setting but requires further validation.

## Acknowledgments

The authors would like to thank the study team, study participants, and collaborators from the University of Makerere, Kampala, Uganda, Ministry of Health, Kenya, Kenyatta National Hospital, Kenya, Kisumu County Hospital, Kenya, and University of Washington, USA.

## Author contributions

**Conceptualization:** Sarah J. Masyuko, John Kinuthia, Alfred O. Osoti, Damalie Nakanjako, Carey Farquhar, Stephanie T. Page.

**Data curation:** Maritza T. Farrant, Sarah J. Masyuko, Jerusha N. Mogaka.

**Formal analysis:** Maritza T. Farrant.

**Funding acquisition:** Sarah J. Masyuko, John Kinuthia, Damalie Nakanjako, Carey Farquhar, Stephanie T. Page.

**Investigation:** Maritza T. Farrant, Sarah J. Masyuko, Jerusha N. Mogaka, Tecla M. Temu, Faith Ameda, Carey Farquhar, Stephanie T. Page.

**Methodology:** Sarah J. Masyuko, Alfred O. Osoti, Tecla M. Temu, Jerry S. Zifodya, Damalie Nakanjako, Faith Ameda, Carey Farquhar, Stephanie T. Page.

**Project administration:** Sarah J. Masyuko, Jerusha N. Mogaka, Carey Farquhar, Stephanie T. Page.

**Resources:** Carey Farquhar, Stephanie T. Page.

**Software:** Maritza T Farrant, Sarah J. Masyuko.

**Supervision:** Sarah J. Masyuko, Tecla M. Temu, Carey Farquhar, Stephanie T. Page.

**Validation:** Sarah J. Masyuko, Alfred O. Osoti, Jerusha N. Mogaka, Tecla M. Temu, Jerry S. Zifodya, Carey Farquhar.

**Visualization:** Maritza T. Farrant, Carey Farquhar, Stephanie T. Page.

**Writing – original draft:** Maritza T. Farrant, Stephanie T. Page.

**Writing – review & editing:** Maritza T. Farrant, Sarah J. Masyuko, John Kinuthia, Alfred O. Osoti, Jerusha N. Mogaka, Tecla M. Temu, Jerry S. Zifodya, Damalie Nakanjako, Faith Ameda, Carey Farquhar, Stephanie T. Page.

## References

[1] Hemkens LG Bucher HC. HIV infection and cardiovascular disease. Eur Heart J. 2014;35:1373–81. 24408888 10.1093/eurheartj/eht528

[2] Islam FM Wu J Jansson J. Relative risk of cardiovascular disease among people living with HIV: a systematic review and meta-analysis. HIV Med. 2012;13:453–68. 22413967 10.1111/j.1468-1293.2012.00996.x

[3] Nou E Lo J Grinspoon SK. Inflammation, immune activation, and cardiovascular disease in HIV. Aids. 2016;30:1495–509. 27058351 10.1097/QAD.0000000000001109 PMC4889507

[4] Stein JH Currier JS Hsue PY. Arterial disease in patients with human immunodeficiency virus infection: what has imaging taught us? JACC Cardiovasc Imaging. 2014;7:515–25. 24831212 10.1016/j.jcmg.2013.08.019 PMC4024182

[5] Klein D Hurley LB Quesenberry CP. Do protease inhibitors increase the risk for coronary heart disease in patients with HIV-1 infection? J Acquir Immune Defic Syndr. 2002;30:471–7. 12154337 10.1097/00126334-200208150-00002

[6] Shah ASV Stelzle D Ken Lee K. Global burden of atherosclerotic cardiovascular disease in people living with HIV systematic review and meta-analysis. Circulation. 2018;138:1100–12. 29967196 10.1161/CIRCULATIONAHA.117.033369 PMC6221183

[7] Roth GA Huffman MD Moran AE. Global and regional patterns in cardiovascular mortality from 1990 to 2013. Circulation. 2015;132:1667–78. 26503749 10.1161/CIRCULATIONAHA.114.008720

[8] Lorenz MW Markus HS Bots ML. Prediction of clinical cardiovascular events with carotid intima-media thickness: a systematic review and meta-analysis. Circulation. 2007;115:459–67. 17242284 10.1161/CIRCULATIONAHA.106.628875

[9] Polak JF Pencina MJ Pencina KM. Carotid-wall intima-media thickness and cardiovascular events. N Engl J Med. 2011;365:213–21. 21774709 10.1056/NEJMoa1012592 PMC3153949

[10] Hunt KJ Sharrett AR Chambless LE. Acoustic shadowing on B-mode ultrasound of the carotid artery predicts CHD. Ultrasound Med Biol. 2001;27:357–65. 11369121 10.1016/s0301-5629(00)00353-7

[11] Boulos NM Gardin JM Malik S. Carotid plaque characterization, stenosis, and intima-media thickness according to age and gender in a large registry cohort. Am J Cardiol. 2016;117:1185–91. 26869392 10.1016/j.amjcard.2015.12.062 PMC5371350

[12] Nambi V Chambless L Folsom AR. Carotid intima-media thickness and presence or absence of plaque improves prediction of coronary heart disease risk. the ARIC (Atherosclerosis Risk In Communities) study. J Am Coll Cardiol. 2010;55:1600–7. 20378078 10.1016/j.jacc.2009.11.075 PMC2862308

[13] Goff DC Lloyd-Jones DM Bennett G. 2013 ACC/AHA guideline on the assessment of cardiovascular risk: a report of the American college of cardiology/American heart association task force on practice guidelines. J Am Coll Cardiol. 2014;63(25 PART B):2935–59. 24239921 10.1016/j.jacc.2013.11.005 PMC4700825

[14] Hulten E Mitchell J Scally J. HIV positivity, protease inhibitor exposure and subclinical atherosclerosis: a systematic review and meta-analysis of observational studies. Heart. 2009;95:1826–35. 19632982 10.1136/hrt.2009.177774

[15] Grunfeld C Delaney JA Wanke C. Preclinical atherosclerosis due to HIV infection: carotid intima-medial thickness measurements from the FRAM study. AIDS. 2009;23:1841–9. 19455012 10.1097/QAD.0b013e32832d3b85 PMC3156613

[16] Masyuko SJ Page ST Kinuthia J. Metabolic syndrome and 10-year cardiovascular risk among HIV-positive and HIV-negative adults: a cross-sectional study. Medicine (Baltim). 2020;99:e20845. 10.1097/MD.0000000000020845 PMC7337552 32629671

[17] Organization WH. STEPwise approach to surveillance (STEPS). Who. Published 2009. Available at: http://www.who.int/chp/steps/en/ [accessed June 17th, 2020].

[18] Ssinabulya I Kayima J Longenecker C. Subclinical atherosclerosis among HIV-infected adults attending HIV/AIDS care at two large ambulatory HIV clinics in Uganda. PLoS One. 2014;9:e89537. 24586854 10.1371/journal.pone.0089537 PMC3938501

[19] Stein JH Korcarz CE Post WS. Use of carotid ultrasound to identify subclinical vascular disease and evaluate cardiovascular disease risk: summary and discussion of the American society of echocardiography consensus statement. Prev Cardiol. 2009;12:34–8. 19301689 10.1111/j.1751-7141.2008.00021.x

[20] Ministry of Health. No Title. Kenya National Guidelines for Cardiovascular Diseases Management. Published 2018. Available at: https://repository.kippra.or.ke/xmlui/handle/123456789/1791?msclkid=339638b0afb011ec8f99e953b8a8b445 [accessed June 17th, 2020].

[21] Alberti KGMM Eckel RH Grundy SM. Harmonizing the metabolic syndrome: a joint interim statement of the international diabetes federation task force on epidemiology and prevention; national heart, lung, and blood institute; American heart association; World heart federation; International. Circulation. 2009;120:1640–5. 19805654 10.1161/CIRCULATIONAHA.109.192644

[22] WHO. Definition and Diagnosis of Diabetes Mellitus and Intermediate Hyperglycemia: report of a WHO/IDF Consultation.; 2006. Available at: http://www.who.int/diabetes/publications/diagnosis_diabetes2006/en/index.html [accessed June 17th, 2020].

[23] Mosepele M Hemphill LC Moloi W. Pre-clinical carotid atherosclerosis and sCD163 among virally suppressed HIV patients in Botswana compared with uninfected controls. PLoS One. 2017;12:e0179994. 28662159 10.1371/journal.pone.0179994 PMC5491105

[24] Tien PC Benson C Zolopa AR. The study of fat redistribution and metabolic change in HIV infection (FRAM): methods, design, and sample characteristics. Am J Epidemiol. 2006;163:860–9. 16524955 10.1093/aje/kwj111 PMC3170407

[25] De Groot E Hovingh GK Wiegman A. Measurement of arterial wall thickness as a surrogate marker for atherosclerosis. Circulation. 2004;109(Suppl 23):III33–8. 15198964 10.1161/01.CIR.0000131516.65699.ba

[26] Nonterah EA Boua PR Klipstein-Grobusch K. Classical cardiovascular risk factors and HIV are associated with carotid intima-media thickness in adults from Sub-Saharan Africa: Findings from H3Africa AWI-Gen Study. J Am Heart Assoc. 2019;8:e011506. 31304842 10.1161/JAHA.118.011506 PMC6662137

[27] Vos AG Barth RE Klipstein-Grobusch K. Cardiovascular disease burden in rural Africa: does HIV and antiretroviral treatment play a role?: baseline analysis of the Ndlovu Cohort Study. J Am Heart Assoc. 2020;9:e013466. 32223395 10.1161/JAHA.119.013466 PMC7428654

[28] Fourie CMT Schutte AE Smith W. Endothelial activation and cardiometabolic profiles of treated and never-treated HIV infected Africans. Atherosclerosis. 2015;240:154–60. 25795556 10.1016/j.atherosclerosis.2015.03.015

[29] Parra S Coll B Aragonés G. Nonconcordance between subclinical atherosclerosis and the calculated Framingham risk score in HIV-infected patients: relationships with serum markers of oxidation and inflammation. HIV Med. 2010;11:225–31. 19845792 10.1111/j.1468-1293.2009.00766.x

[30] De Socio GVL Martinelli C Ricci E. Relations between cardiovascular risk estimates and subclinical atherosclerosis in naïve HIV patients: Results from the HERMES study. Int J STD AIDS. 2010;21:267–72. 20378899 10.1258/ijsa.2009.009165

[31] Hsue Y Lo JC Franklin A. Progression of atherosclerosis as assessed by carotid intima-media thickness in patients with HIV infection. Circulation. 2004;109:1603–8. 15023877 10.1161/01.CIR.0000124480.32233.8A

[32] Putcharoen O Pleumkanitkul S Chutinet A. Comparable carotid intima-media thickness among long-term virologically suppressed individuals with HIV and those without HIV in Thailand. J Virus Erad. 2019;5:23–7. http://www.ncbi.nlm.nih.gov/pubmed/30800422%0Ahttp://www.pubmedcentral.nih.gov/articlerender.fcgi?artid=PMC6362911 [accessed July 2020]. 30800422 PMC6362911

[33] Pacheco AG Grinsztejn B Da Fonseca MDJM. HIV infection is not associated with carotid intima-media thickness in Brazil: a cross-sectional analysis from the INI/ELSA-Brasil study. PLoS One. 2016;11:e0158999. 27391355 10.1371/journal.pone.0158999 PMC4938392

[34] Muiru AN Bibangambah P Hemphill L. Distribution and performance of cardiovascular risk scores in a mixed population of HIV-infected and community-based HIV-uninfected individuals in Uganda. J Acquir Immune Defic Syndr. 2018;78:458–64. 29652762 10.1097/QAI.0000000000001696 PMC6019157

[35] Pacheco AG Grinsztejn B De Jesus M. Traditional risk factors are more relevant than HIV-specific ones for carotid intima-media thickness (cIMT) in a Brazilian cohort of HIV-infected patients. PLoS One. 2015;10:e0117461. 25692764 10.1371/journal.pone.0117461 PMC4333203

[36] Sarfo FS Nichols M Agyei B. Burden of subclinical carotid atherosclerosis and vascular risk factors among people living with HIV in Ghana. J Neurol Sci. 2019;397:103–111. 30599299 10.1016/j.jns.2018.12.026 PMC6368351

[37] Baker JV Henry WK Patel P. Progression of carotid intima-media thickness in a contemporary human immunodeficiency virus cohort. Clin Infect Dis. 2011;53:826–35. 21860012 10.1093/cid/cir497 PMC3174096

[38] Clark SJ Gómez-Olivé FX Houle B. Cardiometabolic disease risk and HIV status in rural South Africa: establishing a baseline. BMC Public Health. 2015;15:135. 25885455 10.1186/s12889-015-1467-1 PMC4335669

[39] Ekoru K Young EH Dillon DG. HIV treatment is associated with a twofold higher probability of raised triglycerides: pooled analyses in 21 023 individuals in sub-Saharan Africa. Glob Heal Epidemiol Genomics. 2018;3:e7. 10.1017/gheg.2018.7 PMC5985947 29881632

[40] Mosepele M Mohammed T Mupfumi L. HIV disease is associated with increased biomarkers of endothelial dysfunction despite viral suppression on long-term antiretroviral therapy in Botswana. Cardiovasc J Afr. 2018;29:155–61. 29771268 10.5830/CVJA-2018-003 PMC6107727

[41] Beltrán LM Rubio-Navarro A Amaro-Villalobos JM. Influence of immune activation and inflammatory response on cardiovascular risk associated with the human immunodeficiency virus. Vasc Health Risk Manag. 2015;11:35–48. 25609975 10.2147/VHRM.S65885 PMC4293933

[42] Temu TM Polyak SJ Zifodya JS. Endothelial dysfunction is related to monocyte activation in antiretroviral-treated people with HIV and HIV-negative adults in Kenya. Open Forum Infect Dis. 2020;7:ofaa425. 33094120 10.1093/ofid/ofaa425 PMC7568437

[43] Temu TM Zifodya JS Polyak SJ. Antiretroviral therapy reduces but does not normalize immune and vascular inflammatory markers in adults with chronic HIV infection in Kenya. AIDS. 2021;35:45–51. 33055570 10.1097/QAD.0000000000002729 PMC7718419

